# An American Text-Book of Surgery

**Published:** 1896-09

**Authors:** 


**An American Text-Book of Surgery.** Edited by WILLIAM W. KEEN, M.D., and J. WILLIAM WHITE, M.D. Second Edition. Pp. xv., 1248. Philadelphia: W. B. Saunders. London: The Rebman Publishing Company, Ltd. 1895.

This text-book of surgery is the joint work of thirteen American surgeons, among whom are found most of the best known names on the other side of the Atlantic. The whole work has been harmonised by the two editors, so that it appears like the effort of a single writer. The entire book (we are told in the preface) has been submitted in proof-sheets to all of the authors for mutual criticism and revision; it may therefore be said to express upon important surgical topics the consensus of opinion of the surgeons who have joined in its preparation.

Many of the illustrations are original, and great care has been taken to credit other authors where illustrations have been borrowed from their works.

The work is designed as a text-book for students and practitioners, and we think it will admirably serve its purpose.

In such a work as this descriptions are necessarily brief, and the book makes no pretence of replacing one of the larger systems of surgery; but for the student who is reading for an examination, or for the practitioner who wishes for a handy work of reference, we know of no book on surgery superior to this one.

Among the more recent introductions of surgery we notice an excellent short account of the principal operations for removing the Gasserian ganglion, including Rose's method and the method recommended by Krause and Hartley. Barker's operation for fractured patella is described and illustrated. Macewen's method of treating thoracic aneurysm by introduction of pins; Wyeth's bloodless method of amputating at the hip- and shoulder-joints; the various operations for radical cure of hernia, including Bassini's, Halsted's, Macewen's, and Ball's; the operation of castration for prostatic hypertrophy are all examples of modern methods of treatment well described in this book.

Chapters are found on diseases of the eye and ear, but these are so incomplete, especially the latter, that we have doubts about the advantage of their introduction. A good, though brief, account of diseases of the brain arising from suppurative disease of the ear is given, and the various operations described.

The surgery of the abdomen is well done, and the illustrations are mostly original and good; but Murphy's button meets with scanty notice and still more scanty praise, and no directions whatever are given for using it. In the operation of gastrostomy we are told that in "all instances in which the patient's strength warrants the performance of the operation in two stages, as was first suggested by Mr. Howse, this method should be practised, as it is much safer than that by which the stomach is opened at once." We take leave to doubt the truth of this; and we still further object to the recommendation given to suture the parietal peritoneum to the edge of the skin incision.

In the chapter on rheumatoid arthritis, we are pleased to see that reference is made to Dr. Spender's observations on certain symptoms that are developed early, before any distinct evidence is afforded of articular changes.

We congratulate the editors upon the success of their first edition, and we can safely prognosticate a continued success for this second edition.

